# α7 nicotinic acetylcholine receptor-mediated neuroprotection against dopaminergic neuron loss in an MPTP mouse model via inhibition of astrocyte activation

**DOI:** 10.1186/1742-2094-9-98

**Published:** 2012-05-24

**Authors:** Yuan Liu, Jun Hu, Jie Wu, Chenlei Zhu, Yujian Hui, Yaping Han, Zuhu Huang, Kevin Ellsworth, Weimin Fan

**Affiliations:** 1 Department of Orthopedics, the First Affiliated Hospital of Nanjing Medical University, Guang Zhou Road 300, Nanjing, 210029, China; 2 Department of Infectious Diseases, the First Affiliated Hospital of Nanjing Medical University, Nanjing, 210029, China; 3 Laboratory of Neurophysiology, Barrow Neurological Institute, St. Joseph’s Hospital and Medical Center, Phoenix, AZ, 85013, USA; 4 University of Pittsburgh School of Medicine, Pittsburgh, PA, 15261, USA

**Keywords:** α7 nicotinic acetylcholine receptor, Parkinson’s disease, Astrocyte, Neuroinflammation, Neuroprotection

## Abstract

****Background**:**

Although evidence suggests that the prevalence of Parkinson’s disease (PD) is lower in smokers than in non-smokers, the mechanisms of nicotine-induced neuroprotection remain unclear. Stimulation of the α7 nicotinic acetylcholine receptor (α7-nAChR) seems to be a crucial mechanism underlying the anti-inflammatory potential of cholinergic agonists in immune cells, including astrocytes, and inhibition of astrocyte activation has been proposed as a novel strategy for the treatment of neurodegenerative disorders such as PD. The objective of the present study was to determine whether nicotine-induced neuroprotection in the 1-methyl-4-phenyl-1,2,3,6-tetrahydropyridine (MPTP) mouse model occurs via α7-nAChR-mediated inhibition of astrocytes.

****Methods**:**

Both *in vivo* (MPTP) and *in vitro* (1-methyl-4-phenylpyridinium ion (MPP^+^) and lipopolysaccharide (LPS)) models of PD were used to investigate the role(s) of and possible mechanism(s) by which α7-nAChRs protect against dopaminergic neuron loss. Multiple experimental approaches, including behavioral tests, immunochemistry, and stereology experiments, astrocyte cell cultures, reverse transcriptase PCR, laser scanning confocal microscopy, tumor necrosis factor (TNF)-α assays, and western blotting, were used to elucidate the mechanisms of the α7-nAChR-mediated neuroprotection.

****Results**:**

Systemic administration of nicotine alleviated MPTP-induced behavioral symptoms, improved motor coordination, and protected against dopaminergic neuron loss and the activation of astrocytes and microglia in the substantia nigra. The protective effects of nicotine were abolished by administration of the α7-nAChR-selective antagonist methyllycaconitine (MLA). In primary cultured mouse astrocytes, pretreatment with nicotine suppressed MPP^+^-induced or LPS-induced astrocyte activation, as evidenced by both decreased production of TNF-α and inhibition of extracellular regulated kinase1/2 (Erk1/2) and p38 activation in astrocytes, and these effects were also reversed by MLA.

****Conclusion**:**

Taken together, our results suggest that α7-nAChR-mediated inhibition of astrocyte activation is an important mechanism underlying the protective effects of nicotine.

## **Background**

Administration of the neurotoxin 1-methyl-4-phenyl-1,2,3,6-tetrahydropyridine (MPTP) to establish various animal models of Parkinson’s disease (PD) has proved to be valuable in the analysis of crucial aspects of this neurodegenerative disease. A metabolite of MPTP in astrocytes, 1-methyl-4-phenylpyridinium ion (MPP^+^), is known to block mitochondrial complex I and to lead to the selective dopaminergic neurodegeneration that is also present in PD [1]. Astrocytes, the most abundant glial cell population, are of neuroectodermal origin, and are thought to be essential for brain homeostasis and neuronal function [2]. Evidence suggests that there is enhanced immunoreactivity of glial fibrillary acidic protein (GFAP) in the striatum and substantia nigra (SN) of patients with PD and in mice treated with MPTP. In the MPTP mouse model, astrocyte activation has been found to be concomitant with neuronal death [3-5].

Studies have shown that after neuronal injury, reactive astrocytes can facilitate neuronal destruction by synthesizing and releasing pro-inflammatory cytokines, which can damage local neurons [2]. Reactive astrocytes can also actively contribute to secondary degeneration after CNS insults or in response to inflammatory signaling cues [6]. Consequently, drugs that can inhibit astrocyte activation and the subsequent inflammatory processes may assist in providing new insights into the intrinsic capacity of the CNS to endure pathogenic insults, and can aid in the identification of molecular targets for therapeutic interventions in a variety of neuroinflammatory and neurodegenerative diseases such as PD [2].

Epidemiologic studies have shown that the prevalence of PD is lower in smokers than in non-smokers. Nicotine, a potent agonist to nicotinic acetylcholine receptors (nAChRs), may exert anti-parkinsonian effects [7]. Recent studies suggest that the ‘nicotinic anti-inflammatory pathway’ may have important clinical implications, as treatment with nicotinic agonists can potentially modulate the production of pro-inflammatory cytokines from immune cells via interactions at α7-containing nAChRs (α7-nAChRs). Thus, α7-nAChRs may serve as a crucial link between inflammation and neurodegeneration in PD and could represent a pharmacological target for potential induction of neuroprotection [8].

In the present study, the main objective was to determine, using *in vivo* and *in vitro* models, whether nicotine, acting at α7-nAChRs, could inhibit astrocyte-mediated neuroinflammation triggered by MPTP/MPP^+^, thus potentially preventing dopaminergic neurodegeneration. The potential anti-inflammatory mechanism(s) and regulation of astrocyte activation by nicotine were further investigated using lipopolysaccharide (LPS), which is the most extensively used glial cell activator because of its induction of inflammatory changes and consequent dopaminergic neurodegeneration [9]. Furthermore, we investigated whether α7-nAChR function is associated with modulation of phosphorylation of extracellular regulated kinase1/2 (Erk1/2) and p38 mitogen-activated protein kinases (MAPKs).

## **Methods**

### **Animals and treatments**

All experiments were carried out in accordance with the National Institutes of Health *Guide for the Care and Use of Laboratory Animals* (publication number 85–23, revised 1985) and the Society for Neuroscience *Guidelines for the Care and Use of Animals in Neuroscience Research*, and were approved by the Institutional Animal Care and Use Committee of Nanjing Medical University [5].

Male C57BL/6 black mice (8 to 10-weeks old, weighing 24 to 28 g) were used. All animals were housed in groups of five per cage under standard laboratory conditions with free access to food and water, constant room temperature (22°C) and humidity (50 to 60 %), and a natural day/night cycle.

Nicotine, methyllycaconitine citrate (MLA), and MPTP were dissolved in sterile saline (0.9 % NaCl). Mice were randomly divided into different groups as described below. All drugs were obtained from Sigma Chemical Co. (St. Louis, MO, USA).

Control mice received saline only. Mice in the treatment group received four intraperitoneal injections of MPTP-HCl 20 mg/kg at 2-hour intervals on the same day. For nicotine treatment, mice were given intraperitoneal injections of nicotine (0.25 or 0.5 mg/kg) five times a day at 2-hour intervals over a 2-week period (1 week before, during, and 1 week after MPTP administration). The nicotine was injected 30 minutes before each MPTP injection. MLA 5.0 mg/kg, used for nicotine antagonism studies, was injected 30 minutes before nicotine administration twice a day for 2 weeks (1 week before, during, and 1 week after MPTP administration) [10,11]. Furthermore, cohorts of mice were treated with nicotine 0.5 mg/kg or MLA 5.0 mg/kg alone five times a day at 2-hour intervals for 2 weeks to observe whether those drugs alone influenced behavioral symptoms, dopaminergic neuron degeneration, and/or astrocyte activation.

### **Behavioral tests**

The effects of MPTP on movement were evaluated by a pole test [3]. Briefly, a cork ball (diameter 25 mm) was fixed to the top of a vertical, wooden rough-surfaced pole (diameter 10 mm, height 500 mm). The mouse was placed head upward on the cork ball, and the following activities were recorded: turning downward on the ball, climbing down the upper half of the pole, climbing down the lower half of the pole. The activities were scored as follows: 3 for recorded times of less than 3 seconds, 2 if less than 6 seconds, and 1 if less than 6 seconds. Results were expressed as the total score.

### **Immunochemistry and stereology**

Animals were anaesthetized using chloral hydrate, and perfused with 0.9% NaCl, followed by cold 4% paraformaldehyde in 0.1 mol/l phosphate buffer (pH 7.4). The brains were dissected out and maintained in 4 % paraformaldehyde overnight. Brains were cryopreserved in 30% sucrose in PBS and stored at −70°C until required. Free-floating sections encompassing the entire midbrain were cut on a cryostat. Sections were processed for tyrosine hydroxylase (TH), GFAP, and Mac-1 (marker for microglia [4]) immunohistochemistry as described below. After incubation for 1 h in 10 % BSA with 0.3% Triton X-100 in 0.01 mol/l PBS, the tissue sections (30 mm) were incubated with primary antibodies overnight at 4°C. The primary antibodies used in this study were a mouse antibody against TH (1:3000, Sigma Chemical Co.), goat antibody against GFAP (1:1000; Millipore Corp., Billerica, MA, USA), and rat anti-mouse Mac-1 polyclonal antibody (1:100, CD11b, AbD; Serotec, Oxford, UK). Immunostaining was visualized by using 3,3′-diaminobenzidine, and sections were then counterstained with hematoxylin [5,6,12].

All cell counts were performed by researchers blinded to the experimental status of the animals. The total number of TH-immunoreactive (IR) neurons, GFAP-IR astrocytes, and Mac-1-IR microglia in the substantia nigra pars compacta (SNpc) were counted from six mice per group using an optical fractionator [13], which is an unbiased method of cell counting that is not affected by either the volume of reference or the size of the counted elements (Stereo Investigator software, Microbrightfield, Colchester, VT, USA). In this method, TH-IR neurons, GFAP-IR astrocytes, and Mac-1-IR microglia were counted in the SNpc of every fourth section (30 mm) throughout the entire extent of the SNpc. Each midbrain section was viewed at low power (×10 objective), and the SNpc was outlined in accordance with the established anatomical landmark. Then, at a random starting point, the number of TH-positive neurons, GFAP-positive astrocytes and Mac-1-IR microglia were counted at high power (×100, oil immersion). To avoid double counting of cells with unusual shapes, each type of cell (TH-IR neurons, GFAP-IR astrocytes and Mac-1-IR microglia) was counted only when its nucleus were optimally visualized, which occurred in only one focal plane. After all the appropriate cells were counted, the total numbers of TH-IR neurons, GFAP-IR astrocytes, and Mac-1-IR microglia in the SNpc were calculated using the formula described by West [13]. Sampling grid dimensions were 120 × 120 x 5 mm (x, y, and z axes, respectively).

### **Astrocyte cell cultures**

Primary cultures of mouse astrocytes were prepared from the midbrain of C57BL/6 black newborn mice 1 to 2 days after birth as previously described [6]. In brief, the mid brain was dissected under sterile conditions and the meninges were carefully removed. Brain tissues were dissociated in 0.25% trypsin (Gibco) for 10 minutes at 37°C. The cell suspension was separated by centrifugation at 240 g for 5 minutes, and the cells were transferred to poly-D-lysine pre-coated cell culture flasks in DMEM containing 10% FCS, 100 U/ml penicillin and 100 μg/ml streptomycin. The cultures were maintained at 37°C in a humidified atmosphere of 5% CO_2_ and 95% air. Before the experiments, analyses showed that over 95% of the cells stained positively for the astrocytic marker GFAP (1:800; Abcam, Cambridge, MA, USA). All experiments were performed after approximately 12 to 15 days in culture.

### **Staining with α-bungarotoxin and confocal microscopy**

Primary cultured astrocytes (as described above) were passaged in six-well tissue-culture plates at 5 × 10^5^ cells per well, and then cultured for 24 hours. Thereafter, cultures were incubated with Alexa Fluor 488-conjugated α-bungarotoxin (2.5 μg/ml^;^ B13422, Invitrogen Corp., OR, USA) at 4°C for 15 minutes. Immediately after incubation, these cells were washed with PBS three times, and then fixed for 15 minutes in 4% paraformaldehyde in PBS at room temperature. After fixation, cells were washed once with PBS and then mounted for viewing under a laser scanning confocal microscope (Meta 710 Laser Scanning Microscope, Carl Zeiss Inc., Thornwood, NY, USA).

### **Reverse transcription-polymerase chain reaction and real-time PCR**

For RNA extraction and reverse transcription (RT)-PCR, total RNA was isolated (RNAiso^TM^ Plus; TaKaRa Biotechnology, Dalian, China) from the SN brain region of mice after drug treatment. First-strand cDNA was synthesized from total RNA using a first-strand cDNA synthesis kit (TaKaRa Biotechnology) in accordance with the manufacturer’s instructions. PCR was performed on the equivalent cDNAs from each sample. Amplification was performed with the primer sets shown in Table 1. The thermal cycling conditions for both sets of primers were 94°C for 5 minutes, followed by 30 cycles of 94°C for 30 seconds, 57°C for 30 seconds, and 72°C for 45 seconds, with a final extension step at 72°C for 10 minutes. The PCR products were then separated in a 3% agarose gel containing ethidium bromide, and analyzed using a gel imaging system (model 3500, Tanon Science and Technology Co., Shanghai, China). Each real-time PCR reaction was carried out in triplicate in a total volume of 20 μl with SYBR Green (Premix Ex Taq^TM^; TaKaRa Biotechnology, Dalian, China) under the following conditions: stage 1, 95°C for 30 seconds (1 cycle); stage 2, 95°C for 5 seconds and 60°C for 20 seconds (40 cycles). Determination of the cycle threshold (Ct) value in a PCR amplification curve was performed using a real-time PCR system (LightCycler; Roche Diagnostics, Basel, Switzerland).

**Table 1:** Primers used for amplification

**Name**	**Genbank accession number**	**Direction**	**Sequence, 5′→3′**	**Size of product bp**
GADPH	NM_008084.2	Forward	TGTGTCCGTCGTGGATCTGA	150
		Reverse	TTGCTGTTGAAGTCGCAGGAG	
α7 subunit	NM 007390.3	Forward	AACCATGCGCCGTAGGACA	172
		Reverse	CTCAGCCACAAGCAGCATGAA	

GADPH, glyceraldehyde-3-phosphate dehydrogenase.

### **Tumor necrosis factor-α assay**

Nicotine, LPS, MPP^+^ and MLA were dissolved in buffered Hank’s buffered salt solution at neutral pH (7.0). All drugs were obtained from Sigma Chemical Co.. Cells were plated onto 12-well plates (1.5 ml, 1 × 10^6^/well), and allowed to adhere for 24 hours at 37°C before being subjected to various treatments. When performing treatments, we used FCS-free DMEM, and the amount of TNF-α in the culture medium was determined 24 hours after treatment using a mouse TNF-α ELISA kit (Beijing 4A Biotech Co. Ltd., Beijing, China) [12].

### **Western blotting**

Cells were collected and homogenized in 200 μl lysis buffer. After incubation for 20 minutes on ice, cell lysates were separated by centrifugation, and the protein concentration in the extracts was determined by the Bradford assay. Proteins in the cell extracts were denatured with SDS sample buffer and separated by 10% SDS-PAGE. Proteins were transferred to nitrocellulose membranes using a wet transfer unit (Miniprotein-III; Bio-Rad Laboratories, Inc., Hercules, CA, USA). The membranes were incubated with 5% BSA dissolved in Tris-buffered saline with Tween 20 (TBS-T, 10 mmol/l Tris–HCl, 150 mmol/l NaCl, and 0.1% Tween 20, pH 7.5)) at room temperature for 1 hour, washed three times, and incubated with different antibodies (Erk1/2, phosphor-Erk1/2, p38 and phospho-p38, 1:1000; Cell Signaling Technology Inc., Beverly, MA USA; α7-nAChRs, 1:300, Santa Cruz Biotechnology Inc., Santa Cruz, CA USA) overnight at 41°C. The membranes were washed three times with TBS-T buffer, and incubated with secondary antibody for 1 hour, followed by four washes in TBS-T. Signal detection was performed with an enhanced chemiluminescence kit [6,12]. The results were scanned using a gel imaging system (GelMax Imager; Ultra-Violet Products Ltd., Upland, CA, USA) and measured using analyzing software (Gel-Pro Analyzer software; Media Cybernetics, Inc., Bethesda, MD, USA).

### **Statistical analyses**

All values are expressed as mean ± standard error of the mean (SEM). Differences between means were analyzed using one-way or two-way ANOVA with time and treatment as the independent factors. When ANOVA showed significant differences, pairwise comparisons between means were further analyzed using the Newman-Keuls *post hoc* test. In all analyses, significance was set at *P* = 0.05 [3,5].

## **Results**

### **Nicotine alleviates 1-methyl-4-phenyl-1,2,3,6-tetrahydropyridine-induced deficits in motor coordination**

**Figure 1:**
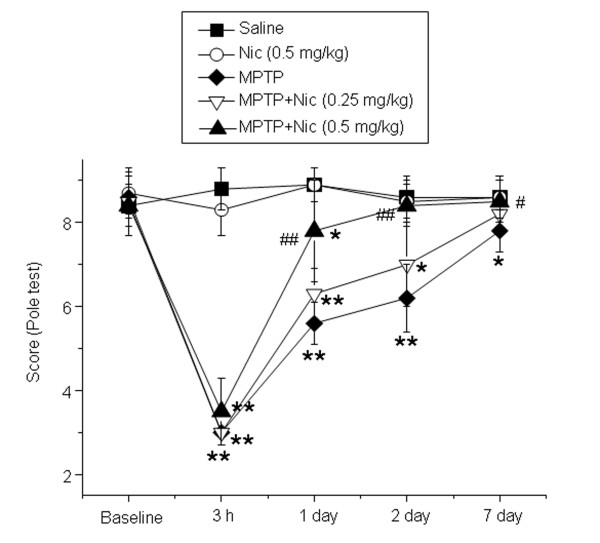
**The effects of nicotine on 1-methyl-4-phenyl-1,2,3,6-tetrahydropyridine (MPTP)-induced mouse motor deficits.** Pole-test scores for each animal were calculated after acute MPTP treatment at 3 hours, 1 day, 2 days and 7 days. Data are presented as mean ± SEM (n = 10). *P < 0.05 and **P < 0.01 versus the control (saline alone) group; ^#^*P* < 0.05 and ^##^*P* < 0.01 versus the MPTP treatment group.

To assess the effects of nicotine on motor function in mice, we used the pole test. Mice were examined under baseline conditions and at 3 hours, 1 day, 2 days and 7 days after MPTP injection. Deficits in motor coordination clearly were seen in MPTP-treated mice at 3 hours, 1 day and 2 days after drug administration. On day 7, there was partial recovery of impaired motor function. Administration of nicotine 0.5 mg/kg significantly increased the performance scores of MPTP-treated mice on days 1, 2 and 7 compared with mice treated with MPTP alone. In particular, on day 2, mice treated with both MPTP and nicotine achieved similar pole-test scores to those of saline-treated control mice, suggesting that nicotine alleviated the MPTP-induced deficits in motor coordination. There was no significant difference in motor performance of mice treated with saline compared with those receiving nicotine alone (Figure 1).

### **Nicotine acting via α7-nicotinic acetylcholine receptors prevents 1-methyl-4-phenyl-1,2,3,6-tetrahydropyridine-induced dopaminergic neuron loss in the substantia nigra pars compacta**

**Figure 2:**
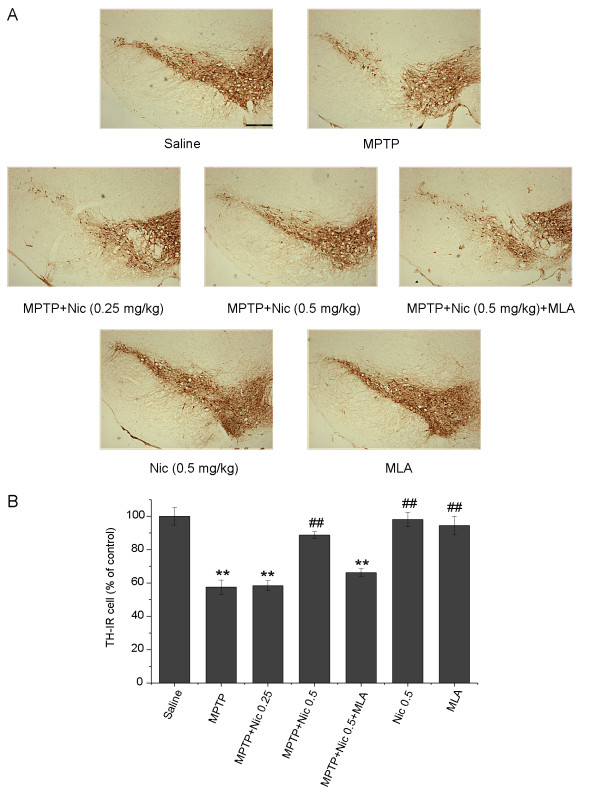
**The effects of nicotine on 1-methyl-4-phenyl-1,2,3,6-tetrahydropyridine (MPTP)-induced dopaminergic neuronal loss in the mouse substantia nigra pars compacta (SNpc). (A)** Tyrosine hydroxylase-immunoreactive (TH-IR) neurons in the mouse SNpc. Scale bar: 250 μm. **(B)** Stereological cell counts of TH-IR neurons in the mouse SNpc. Data are presented as a percentage of controls (mean ± SEM) from four to six mice per group. **P < 0.01 versus the control (saline-only) group; ^##^*P* < 0.01 versus the MPTP treatment group.

Stereological counts of TH-IR cells in the SNpc were undertaken to explore the effects of nicotine on MPTP-induced degeneration of dopaminergic neurons. Dopaminergic neurons in the SNpc were intensely immunoreactive to TH in control (saline-only) mice (Figure 2A). Treatment with MPTP significantly reduced the numbers of TH-IR neurons by 42.5 ± 4.2% compared with controls. However, in mice treated with both MPTP and nicotine 0.5 mg/kg, dopaminergic neurons were reduced by only 11.3 ± 2.0% compared with controls (*P* > 0.05). The difference in the numbers of TH-IR neurons between mice treated with MPTP alone and those treated with both MPTP and nicotine was significant (*P* < 0.01). Systemic administration of nicotine alone did not significantly alter the numbers of TH-IR neurons (*P* > 0.05 compared with controls). To determine whether the protective effect of nicotine against MPTP-induced dopaminergic neuronal degeneration was mediated via α7-nAChRs, mice were pretreated with the α7-nAChR-selective antagonist MLA 5.0 mg/kg, 30 minutes before nicotine administration. MLA significantly reversed the protective effect of nicotine, as evidenced by a reduction in the numbers of dopaminergic neurons by 33.8 ± 2.3% compared with controls (*P* < 0.01; Figure 2B). There were significant differences between the MLA pretreatment group and the group treated with both MPTP and nicotine (*P* < 0.01), but not between the MLA pretreatment group and the group treated with MPTP alone (*P* > 0.05). MLA administration alone did not significantly affect the numbers of TH-IR neurons (*P* > 0.05 compared with controls) (Figure 2A,B).

### **Nicotine acting via α7-nicotinic acetylcholine receptors inhibits 1-methyl-4-phenyl-1,2,3,6-tetrahydropyridine-induced astrocyte activation in the substantia nigra pars compacta**

**Figure 3:**
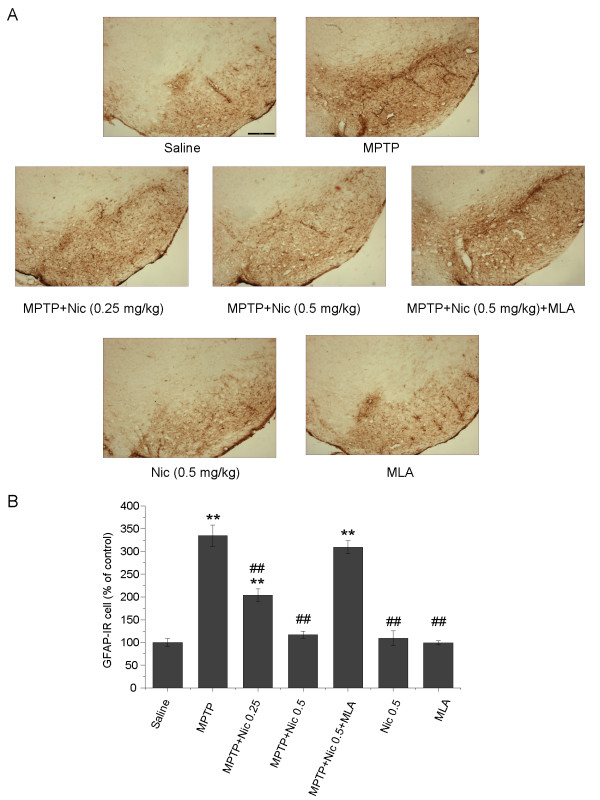
**The effects of nicotine on 1-methyl-4-phenyl-1,2,3,6-tetrahydropyridine (MPTP)-induced astrocyte activation in the mouse substantia nigra pars compacta (SNpc). (A)** Glial fibrillary acidic protein immunoreactive (GFAP-IR) astrocytes in mouse SNpc. Scale bar: 250 μm. **(B)** Stereological cell counts of GFAP-IR astrocytes in the mouse SNpc. Data are presented as a percentage of controls (mean ± SEM) from four to six mice per group. **P < 0.01 versus the control (saline-only) group; ^##^*P* < 0.01 versus the MPTP treatment group.

To evaluate the effects of nicotine on astrocyte activation, immunostaining was used to detect the astrocyte marker GFAP. Acute administration of MPTP induced marked astrocyte activation in the mouse SN (Figure 3A,B), as evidenced both by a significant increase in the numbers of GFAP-IR cells (334.6 ± 23.9% increase compared with controls) and by morphological changes such as larger cell bodies and longer hypertrophic processes (data not shown). MPTP-induced astrocyte activation and corresponding increases in the number of GFAP-IR cells were suppressed by the addition of nicotine. Specifically, nicotine at concentrations of 0.25 and 0.5 mg/kg suppressed MPTP-induced increases in GFAP-IR cells by 38.9 ± 5.6% and 65.0 ± 3.1%, respectively (*P* < 0.01) compared with MPTP treatment alone. No significant increases in the numbers of GFAP-IR cells were seen in mice treated with nicotine alone (*P* > 0.05 compared with controls). Interestingly, the protective effect of nicotine against astrocyte activation was reversed by pretreatment with MLA 5.0 mg/kg (Figure 3A,B). MLA significantly reversed nicotinic inhibition of astrocyte activation by increasing the numbers of GFAP-IR cells to 309.6 ± 14.2% compared with controls (*P* < 0.01). There were significant differences between the MLA pretreatment group and the group treated with both MPTP and nicotine (*P* < 0.01), but there were no significant differences between the MLA pretreatment group and the group treated with MPTP alone (*P* > 0.05). MLA administration alone did not significantly affect the numbers of GFAP-IR cells (*P* > 0.05 compared with controls) (Figure 3A,B). These results suggest that nicotine probably inhibits MPTP-induced astrocyte activation via its actions at α7-nAChRs.

### **Nicotine acting via α7-nicotinic acetylcholine receptors inhibits 1-methyl-4-phenyl-1,2,3,6-tetrahydropyridine-induced microglia activation in the substantia nigra pars compacta**

**Figure 4:**
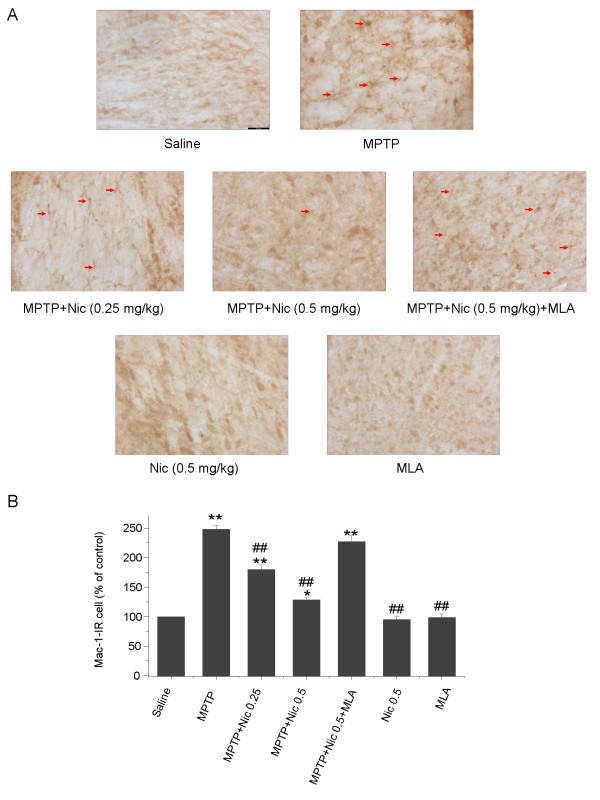
**The effects of nicotine on 1-methyl-4-phenyl-1,2,3,6-tetrahydropyridine (MPTP)-induced microglia activation in the mouse substantia nigra pars compacta (SNpc).(A) Mac-1-IR microglia in mouse SNpc. Scale bar: 200 μm.(B)** Stereological cell counts of Mac-1-IR microglia in the mouse SNpc. Data are presented as a percentage of controls (mean ± SEM) from four to six mice per group. *P < 0.05 and **P < 0.01 versus the control (saline-only) group; ^##^*P* < 0.01 versus the MPTP treatment group.

To evaluate the effects of nicotine on microglia activation, immunostaining was used to detect the microglia marker Mac-1. Acute administration of MPTP induced marked microglia activation in the mouse SN (Figure 4A,B), as evidenced both by a significant increase in the numbers of Mac-1-IR cells (247.9 ± 6.6% increase compared with controls) and by the corresponding cellular morphological changes such as larger cell bodies and poorly ramified short thick processes. The MPTP-induced microglia activation and corresponding increases in the numbers of Mac-1-IR cells were suppressed by the addition of nicotine. Specifically, nicotine, at concentrations of 0.25 and 0.5 mg/kg, suppressed MPTP-induced increases in Mac-IR cells by 27.5 ± 7.8% and 48.3 ± 9.4%, respectively (*P* < 0.01), compared with MPTP treatment alone. No significant increases in the numbers of Mac-1-IR cells were seen in mice treated with nicotine alone (*P* > 0.05 compared with controls). Interestingly, the protective effect of nicotine against microglia activation was reversed by pretreatment with MLA 5.0 mg/kg (Figure 4A,B), which resulted in an increase in the numbers of Mac-1-IR cells by 227.0 ± 9.8 % compared with controls (*P* < 0.01). There were significant differences between the MLA pretreatment group and the group treated with both MPTP and nicotine (*P* < 0.01), but not between the MLA pretreatment group and the group treated with MPTP alone (*P* > 0.05). MLA administration alone did not significantly affect the numbers of Mac-1-IR cells (*P* > 0.05 compared with controls) (Figure 4A,B). These results suggest that nicotine probably inhibits MPTP-induced microglia activation via its actions at α7-nAChRs.

### **Expression of the α7-nicotinic acetylcholine receptor subunit in mouse astrocytes**

**Figure 5:**
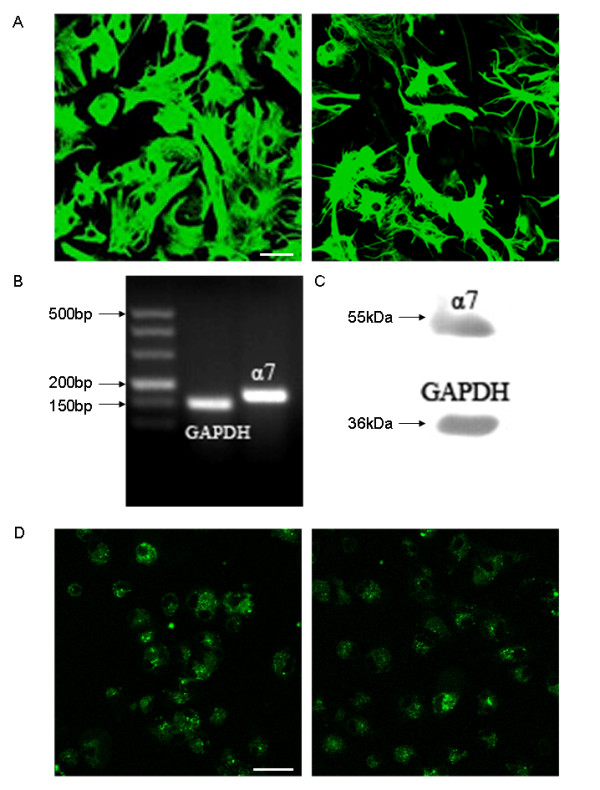
**Expression of the α7-nicotinic acetylcholine receptor (α7-nAChR) subunit expression in primary astrocyte cell cultures. (A)** Glial fibrillary acidic protein (GFAP) immunofluorescence staining of astrocytes in primary cultured astrocyte cells from the midbrain of C57BL/6 black newborn mice 1–2 days after birth. Scale bar: 50 μm. **(B)** Reverse transcription (RT)-PCR analysis of α7 subunit mRNA expression in primary cultured astrocyte cells. **(C)** Western blot of α7 nAChR protein in primary cultured astrocyte cells. **(D)** Primary mouse astrocytes stained with Alexa Fluor 488 conjugate-labeled α-bungarotoxin and viewed under fluorescence confocal microscopy. Scale bar: 50 μm.

To further test the hypothesis that nicotine-induced neuroprotection in an MPTP mouse model is mediated through its actions at α7-nAChRs on astrocytes, the expression of α7-nAChRs was evaluated using primary cultured astrocytes. Cell analyses showed that more than 95 % of primary cultured mouse astrocytes were GFAP-positive (Figure 5A). To determine whether α7-nAChR subunits were expressed in astrocytes, total RNA from primary cultured astrocytes was first isolated for RT–PCR and real-time PCR analyses. The results showed that α7-nAChR subunit mRNA was detected in astrocytes (Figure 5B). The Ct of glyceraldehyde-3-phosphate dehydrogenase and α7-nAChR were 11.6 ± 0.2 and 26.6 ± 0.8, respectively. Fluorescence signals from cells entering the exponential growth phase gave positive results. Furthermore, the α7-nAChR subunit protein was detected by western blotting in primary cultured astrocytes (Figure 5C). To evaluate whether α-bungarotoxin, an α7-nAChR subunit-selective blocker, could bind to this receptor, we stained primary astrocytes with Alexa Fluor 488 conjugate-labeled α-bungarotoxin. Strong binding of α-bungarotoxin was seen on the astrocyte surface (Figure 5D).

### **Nicotine acting via α7-nicotinic acetylcholine receptors decreases 1-methyl-4-phenylpyridinium ion- or lipopolysaccharide-induced production of tumor necrosis factor-α from mouse primary cultured astrocytes**

**Figure 6:**
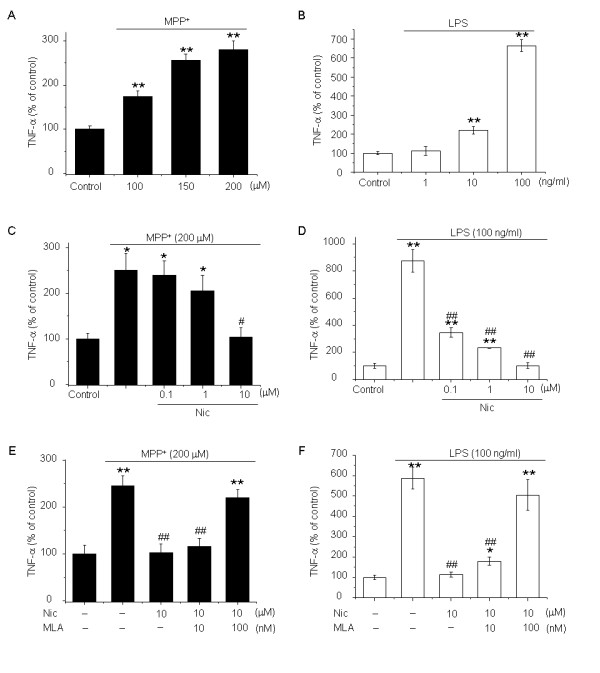
**The effects of nicotine on enhanced production from astrocytes of tumor necrosis factor (TNF)-α induced by 1-methyl-4-phenylpyridinium ion (MPP**^**+**^**) and lipopolysaccharide (LPS). (A)** MPP^+^ and **(B)** LPS increased the production of TNF-α in astrocytes. Various concentrations of nicotine suppressed the otherwise enhanced release of TNF-α induced by **(C)** 200 μmol/l MPP^+^ or **(D)**100 ng/ml LPS in astrocytes. The α7-nAChR-selective antagonist methyllycaconitine (MLA) abolished the inhibitory effects of nicotine on the release of TNF-α induced by **(E)** MPP^+^ and **(F)** LPS. Data are presented as a percentage of controls (mean ± SEM) of four independent experiments. *P < 0.05 and **P < 0.01 versus the control group; ^#^*P* < 0.05 and ^##^*P* < 0.01 versus the MPP^+^ treatment alone group

The data presented thus far suggest that nicotine, probably acting at α7-nAChRs, protects dopaminergic neurons in the mouse SNpc from the effects of acute MPTP treatment. One potential mechanism underlying the protective effect of nicotine is that activation of α7-nAChRs suppresses astrocyte activation and in turn decelerates or eliminates neuronal inflammatory responses. To test this hypothesis, we evaluated the effects of nicotine on release of the inflammatory factor TNF-α induced by MPP^+^ or the bacterial endotoxin LPS in cultured mouse astrocytes. Incubation of cultured mouse astrocytes with MPP^+^ 200 μmol/l or LPS 100 ng/ml for 24 hours significantly increased the production of TNF-α (*P* < 0.01 versus control) (Figure 6A,B). Notably, MPP^+^ 200 μmol/l or LPS 100 ng/ml induced astrocyte activation but failed to affect astrocyte viability (data not shown). Pretreatment with nicotine (0.1, 1 or 10 μmol/l) for 30 minutes before the addition of MPP^+^ or LPS decreased the production of TNF-α in a concentration-dependent manner (Figure 6C,D). Specifically, nicotine 10 μmol/l reduced MPP^+^- and LPS-induced production of TNF-α by 58.0 ± 6.7 % (*P* < 0.05 versus MPP^+^ treatment alone) and 88.5 ± 1.7 % (*P* < 0.01 versus LPS treatment alone), respectively (Figure 6C,D). Preincubation with MLA 100 nmol/l for 30 minutes before administration of nicotine fully reversed its inhibitory effects (Figure 6E,F). There were no significant differences between the MLA pretreatment group and the groups treated with MPP^+^ or LPS alone. These results suggest that nicotine significantly abolishes MPP^+^- or LPS-induced production of pro-inflammatory factors from astrocytes via stimulation of α7-nAChRs.

### **Nicotine acting via α7-nicotinic acetylcholine receptors suppresses extracellular regulated kinase1/2 and p38 mitogen-activated protein kinase activation induced by 1-methyl-4-phenylpyridinium ion in astrocytes**

**Figure 7:**
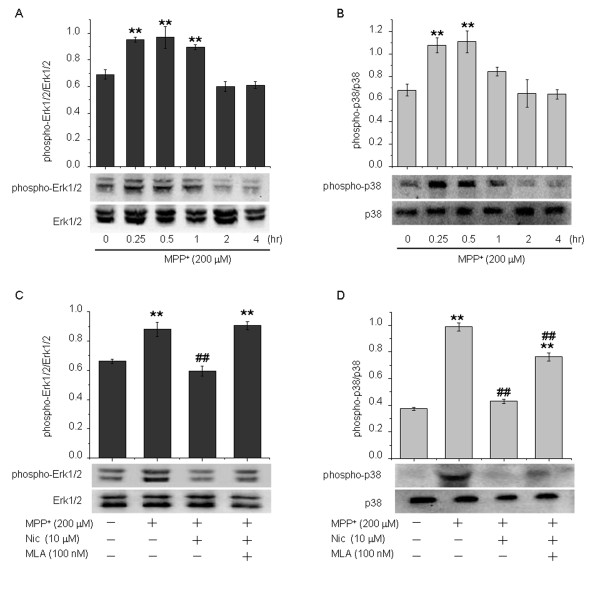
**The effects of nicotine on activation of extracellular regulated kinase (Erk)1/2 and p38 mitogen-activated protein kinase (MAPK) induced by 1-methyl-4-phenylpyridinium ion (MPP**^**+**^**) in astrocytes.** MPP^+^ increased Erk1/2 and p38 MAPK phosphorylation indicative of their activation. **(A,B)** Astrocytes were treated with MPP^+^ 200 μmol/l for various lengths of time as indicated.**(C,D)** Astrocytes were pretreated with nicotine 10 μmol/l in the absence or presence of MLA 100 nmol/l for 30 minutes and then challenged with MPP^+^ 200 μmol/l for 30 minutes. After treatment, astrocytes were harvested, and levels of phosphorylated (A,C) Erk1/2 and (B,D) p38 MAPKs were analyzed. Upper panel shows densitometric analysis of the phosphorylated forms of Erk1/2 and p38 MAPK, while the lower panel shows representative blots. Data are presented as the mean ± SEM of four independent experiments. *P < 0.05 and **P < 0.01 versus the control group; ^#^*P* < 0.05 and ^##^*P* < 0.01 versus the MPP^+^ treatment alone group.

MAPKs collectively constitute key signaling molecules in processes of neuroinflammation. Phosphorylation of MAPKs leads to the initiation of signal cascades that regulate the synthesis of a variety of pro-inflammatory factors such as TNF-α in microglia [12] and astrocytes [6]. In the present study, we investigated whether nicotine eliminated the MPP^+^-induced phosphorylation of both Erk1/2 and p38 MAPKs in astrocyte cultures. Treatment with MPP^+^ 200 μmol/l induced rapid and transient phosphorylation of both Erk1/2 and p38, reflecting their activation, and peak levels of phosphorylated Erk1/2 and p38 occurred after 30 minutes (Figure 7A,B). Levels of phosphorylated Erk1/2 and p38 were sustained for up to 1 hour after MPP^+^ treatment. These results suggest that both Erk1/2 and p38 MAPKs are activated in response to MPP^+^ stimulation in astrocytes. Next, we explored the effects of nicotine on MPP^+^-induced Erk1/2 and p38 MAPK phosphorylation. Pretreatment with 10 μmol/l nicotine suppressed MPP^+^-induced increases in the levels of phosphorylated Erk1/2 and p38 by 32.3 ± 1.7 % (*P* < 0.01) and 56.4 ± 3.1 % (*P* < 0.01), respectively (Figure 7C,D). Moreover, the suppressive effects of nicotine on Erk1/2 phosphorylation were completely reversed by 30 minutes of pretreatment with MLA 100 nmol/l. Interestingly, the suppressive effects of nicotine on p38 phosphorylation were only partially reversed by MLA. These data suggest that nicotine acting at α7-nAChRs suppresses MAPK signaling transduction, thereby affecting processes mediating neuroinflammation.

### **Nicotine acting via α7-nicotinic acetylcholine receptors suppresses lipopolysaccharide-induced extracellular regulated kinase1/2 and p38 mitogen-activated protein kinase activation in astrocytes**

**Figure 8:**
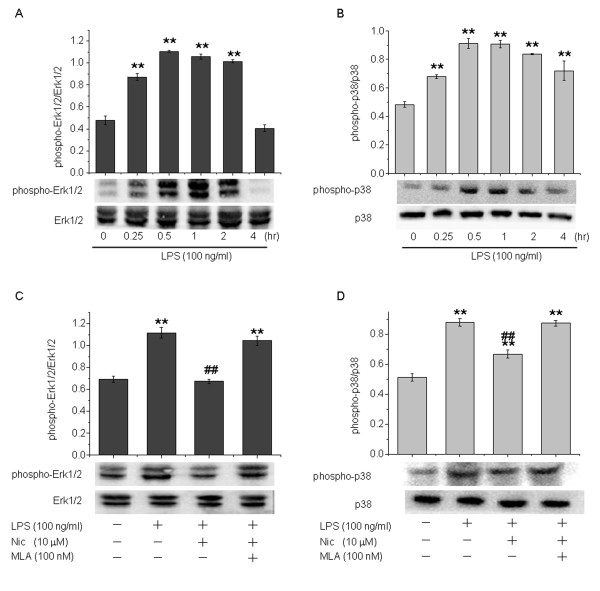
**The effects of nicotine on lipopolysaccharide (LPS)-induced Erk1/2 and p38 mitogen-activated protein kinase (MAPK) activation in astrocytes.** LPS also increased Erk1/2 and p38 MAPK phosphorylation, reflecting their activation. **(A,B)** Astrocytes were treated with LPS 100 ng/ml for various lengths of time as indicated. **(C,D)** Astrocytes were pretreated with nicotine 10 μmol/l in the absence or presence of MLA 100 nmol/l for 30 minutes and then challenged with LPS for 30 minutes. After treatment, astrocytes were harvested, and levels of phosphorylated **(A,C)** Erk1/2 and **(B,D)** p38 MAPK were analyzed. Upper panel shows densitometric analysis of the phosphorylated forms of Erk1/2 and p38 MAPK, while the lower panel shows representative blots.. Data are presented as the mean ± SEM of four independent experiments. *P < 0.05 and **P < 0.01 versus the control group; ^#^*P* < 0.05 and ^##^*P* < 0.01 versus the LPS treatment alone group.

LPS has been extensively used as a glial activator for the induction of inflammatory dopaminergic neurodegeneration [9]. We examined whether nicotine also regulated LPS-induced MAPK signaling transduction. Astrocytes were treated with LPS 100 ng/mL for different lengths of times to determine the extent of MAPK activation after LPS stimulation. Treatment with LPS also led to rapid and transient phosphorylation of both Erk1/2 and p38, with peak levels similarly occurring at 30 minutes (Figure 8A,B). Levels of phosphorylated Erk1/2 and p38 were sustained for up to 2 and 4 hours, respectively, after LPS administration. These results suggest that both Erk1/2 and p38 are activated in response to LPS stimulation in astrocytes. We then investigated whether nicotine could regulate LPS-induced Erk1/2 and p38 phosphorylation. Pretreatment with nicotine 10 μmol/l suppressed LPS-induced increases in phosphorylated Erk1/2 and p38 by 39.6 ± 2.3 % (*P* < 0.01) and 23.8 ± 5.1 % (*P* < 0.01), respectively (Figure 8C,D). Moreover, the suppressive effects of nicotine on Erk1/2 and p38 phosphorylation were completely reversed by MLA (100 nmol/l). These data suggest that nicotine, through its actions at α7-nAChRs, also inhibits LPS-induced astrocyte activation via suppression of MAPK signaling transduction.

## **Discussion**

In the brain, two types of glial cells, astroglia and microglia, are the main players in neuroinflammatory processes. Microglia, which are resident immune cells within the brain, are considered to be ‘professional’ macrophages of the CNS. For this reason, most of the studies of neuroinflammatory processes in the CNS have focused on microglia. The contribution of astroglia to these processes often has been neglected. However, recent evidence suggests that astroglia, which represent the most abundant glial cell population, mediate crucial neuroinflammatory processes involved in the initiation and subsequent development of neurodegenerative disorders such as PD [2]. Our results and those obtained from previous studies identified enhanced immunoreactivity of GFAP-labeled astrocytes in the SN of MPTP-treated mice, suggesting that reactive astrocytes may play a key role in brain regions damaged by MPTP [3-5]. Thus, drugs that can regulate astrocyte activation may potentially exert neuroprotective effects against MPTP/MPP^+^-induced toxicity.

In the present study, we found that systemic administration of nicotine significantly alleviated MPTP-induced perturbed behavioral symptoms, improved compromised motor coordination, protected against degeneration of dopaminergic neurons, and inhibited activation of astrocytes and microglia in the SN. Furthermore, we found that nicotine suppressed MPP^+^- or LPS-stimulated elevation of TNF-α released from reactive astrocytes by downregulating phosphorylation of Erk1/2 and p38 MAPKs. Erk1/2 [14] and p38 [15] MAPK pathways were originally identified as signaling cascades activated by pro-inflammatory stimuli and cellular stresses, and they have been shown to play crucial roles in translational regulation of pro-inflammatory cytokine synthesis. Previous studies have shown that inhibition of Erk1/2 and p38 MAPKs reduced expression of inducible nitric oxide synthase (iNOS) and cyclooxygenase-2 mRNA, suppressed the production of several pro-inflammatory cytokines including TNF-α, and rescued dopaminergic neurons in the SN [16]. Taken collectively, our data suggest that the Erk1/2 and p38 MAPK signaling-transduction pathways are involved in the inhibitory effects of nicotine on MPP^+^- or LPS-induced astrocyte activation.

Interestingly, it has been suggested that MPP^+^ induces astrocytic apoptosis via regulation of MAPK signaling [17]. Increasing evidence has shown that astrocytic apoptosis may contribute to the pathogenesis of many neurodegenerative disorders such as Alzheimer’s disease and PD [18]. Based upon our findings that nicotine decreased Erk1/2 and p38 MAPK activation in reactive astrocytes, further studies are needed to evaluate the potential role(s) of nicotine in the regulation of astroglial apoptosis.

Epidemiological studies [19] have reported that the prevalence of PD is lower in smokers than in non-smokers, leading to the hypothesis that the reduced prevalence of PD in smokers may be due to the presence of nicotine in tobacco. Nicotine administration has been reported to improve motor deficits that arise from nigrostriatal damage in parkinsonian animals and in patients with PD. In addition, nicotine has been shown to exert protective effects against nigrostriatal damage in various animal models of PD [20,21]. Although these observations suggest that nicotine may be beneficial for the treatment of PD, the specific mechanisms underlying the potentially protective effects of nicotine remain obscure. For example, nicotine may play a neuroprotective role via modulation of both monoamine oxidase activity and complex I of the electron transport chain [7]. The development of studies focused on the neuroprotective effects exerted by specific nAChR subtypes would be beneficial, because their identification and the description of their potential roles in the pathogenesis of PD might facilitate the development of targeted therapies. The nicotine-induced neuroprotective effects that have been shown in many animal models might be mediated via a number of nAChR subtypes, including but not limited to α4β2 [22], α7 [21], α6α4β2 [23], and α6β2 [24].

Our results showing that nicotine protected against the loss of dopaminergic neurons in the MPTP-induced mouse model of PD are consistent with those of several previous reports [21,25]. However, the mechanisms underlying nAChR-mediated neuroprotection remain unknown. nAChRs are transmitter-gated ion channels, and nine different α subunits (α2 to α10) and three β subunits (β2 to β4) have been cloned to date [26]. Different combinations of α and β subunits, or of α subunits alone, produce various receptor subtypes that exhibit different physiologic, pharmacologic, and anatomic properties. Recently, the α7 subunit has received considerable attention because studies have suggested that it plays a role in cholinergic anti-inflammatory processes [27]. Specifically, mounting evidence has shown that nicotine inhibits inflammatory signaling and the consequent production of relevant pro-inflammatory cytokines via its actions at α7-nAChRs [14,28-30].

In the present study, we found that the alleviation of MPTP-induced loss of dopaminergic neurons in the SNpc and inhibition of astrocyte and microglia activation in the SN by nicotine *in vivo* was reversed by the α7-nAChR-selective antagonist MLA. Interestingly however, MLA did not reverse the alleviation by nicotine of MPTP-induced behavioral symptoms (data not shown). A more extensive evaluation system should be used to elucidate more clearly mouse behavior in models of PD [31]. Additionally, in mouse primary cultured astrocytes, pretreatment with nicotine suppressed MPP^+^- or LPS-induced astrocyte activation, as evidenced by both decreased production of TNF-α and inhibition of Erk1/2 and p38 MAPK activation. These inhibitory effects of nicotine were also reversed by MLA.

The primary cascade activated by the neurotoxin MPTP in mice might differ depending upon whether treatment is acute or chronic [32,33]. Liberatore *et al*. [4] reported that mutant mice lacking the iNOS gene were significantly more resistant to acute MPTP treatment compared with wild-type littermates in an experiment conducted to investigate the mechanisms of protection against inflammation. Furuya *et al*. [32] examined the role of caspase-11 in inflammation and apoptosis immediately after MPTP administration in caspase-11 knockout mice and in their wild-type littermates, and results indicated that the inflammatory pathway seems to be closely related to the animal model for acute MPTP treatment. Therefore, we used an acute MPTP model to investigate the mechanisms of α7-nAChR-mediated neuroprotection against inflammation. There may be changes in receptor kinetics secondary to desensitization and/or possible receptor downregulation after prolonged nicotine exposure, thus our findings need to be confirmed using *in vivo* experiments with chronic exposure, which more closely resembles the conditions that occur during smoking or under therapy with nicotine or nicotinic ligands.

## **Conclusions**

This is the first report that nicotine inhibits MPTP (*in vivo*)- and MPP^+^- or LPS (*in vitro*)-induced astrocyte activation via its actions at α7-nAChRs and decreases the consequent production of pro-inflammatory factors, thus alleviating dopaminergic neurodegeneration. Our results strongly support the hypothesis that inhibition of astrocyte activation by stimulation of α7-nAChRs may provide a new therapeutic strategy for treatment of neuroinflammation-related disorders. Thus, nicotinic agonists that specifically target the α7-nAChR might serve as potential therapeutic agents for PD and other neuroinflammation-related neurodegenerative diseases.

## **Abbreviations**

α7-nAChR, α7-nicotinic acetylcholine receptor; BSA, Bovine serum albumin; CNS, Central nervous system; DMEM, Dulbecco’s modified Eagle’s medium; ELISA, Enzyme-linked immunosorbent assay; Erk, Extracellular regulated kinase; FCS, Fetal calf serum; GFAP, Glial fibrillary acidic protein; IR, Immunoreactive; LPS, Lipopolysaccharide; MAPK, Mitogen-activated protein kinase; MLA, Methyllycaconitine; MPP+, 1-methyl-4-phenylpyridinium ion; MPTP, 1-methyl-4-phenyl-1,2,3,6-tetrahydropyridine; PBS, Phosphate buffered saline; PD, Parkinson’s disease; SNpc, Substantia nigra pars compacta; RT, reverse transcripton; SDS-PAGE, Sodium dodecyl sulfate polyacrylamide gel electrophoresis; TBS-T, Tris-buffered saline with Tween 20; TH, Tyrosine hydroxylas; TNF, tumor necrosis factor.

## **Competing interests**

The authors declare that they have no competing interests.

## **Authors’ contributions**

JH and WF designed the experimental protocols, which were carried out by YL, JW, CZ, YH, YH, ZH, KE and JH. The manuscript was prepared by YL, JW, KE and JH. All authors read and approved the final manuscript.
